# PLD1 promotes dendritic spine development by inhibiting ADAM10-mediated N-cadherin cleavage

**DOI:** 10.1038/s41598-017-06121-2

**Published:** 2017-07-20

**Authors:** Li-Da Luo, Gang Li, Yun Wang

**Affiliations:** 1 Neuroscience Research Institute and Department of Neurobiology, School of Basic Medical Sciences, Key Laboratory for Neuroscience, Ministry of Education/National Health and Family Planning Commission, Peking University, Beijing, 100191 China; 2 PKU-IDG/McGovern Institute for Brain Research, Peking University, Beijing, 100871 China

## Abstract

Synapses are the basic units of information transmission, processing and integration in the nervous system. Dysfunction of the synaptic development has been recognized as one of the main reasons for mental dementia and psychiatric diseases such as Alzheimer’s disease and autism. However, the underlying mechanisms of the synapse formation are far from clear. Here we report that phospholipase D1 (PLD1) promotes the development of dendritic spines in hippocampal neurons. We found that overexpressing PLD1 increases both the density and the area of dendritic spines. On the contrary, loss of function of PLD1, including overexpression of the catalytically-inactive PLD1 (PLD1ci) or knocking down PLD1 by siRNAs, leads to reduction in the spine density and the spine area. Moreover, we found that PLD1 promotes the dendritic spine development via regulating the membrane level of N-cadherin. Further studies showed that the regulation of surface N-cadherin by PLD1 is related with the cleavage of N-cadherin by a member of the disintegrin and metalloprotease family-ADAM10. Taking together, our results indicate a positive role of PLD1 in synaptogenesis by inhibiting the ADAM10 mediated N-cadherin cleavage and provide new therapeutic clues for some neurological diseases.

## Introduction

With the enrichment of different kinds of receptors for neural transmission, dendritic spines are important parts for information processing and integration^1, 2^. The dendritic spines must connect precisely with presynaptic terminals in order to match with each other correctly and thus constitute properly functional synapses. Moreover, the connections between presynaptic and postsynaptic parts need appropriate regulation to ensure that the synapses stay reliable and balanced. In accordance with this, a majority of neurological diseases are accompanied with the aberrant development of dendritic spines^3–5^. Particularly, most of the genes which are highly correlated with psychiatric diseases also play important roles in dendritic spine development^6–11^. Thus, elucidating the mechanisms of dendritic spine development is crucial for understanding both the assembly of neural connections and the pathology for neurological diseases.

With the accumulation of the understandings for the mechanisms underlying synapse formation, it has been widely accepted that lipid plays an important role in neuronal morphorgenesis^12–14^. Accordingly, the homeostasis of lipid components is related to both the intellectual development and the mental disorders such as anxiety and depression^15–17^. For a neuronal cell, phospholipids are important components for biological membrane systems, among which phosphatidylcholine (PC) represents the highest level^18^. PC has been reported to be involved in neural differentiation^19^, learning and memroy^20^, sleep^21^, and Alzheimer’s diseases^22, 23^, prompting critical roles of the PC-regulating enzymes in the nervous system.

Phospholipase D1 (PLD1) which is responsible for catalyzing the hydrolysis of PC into phosphatidic acid (PA) and choline, has recently been reported to participate in neuronal signaling^24, 25^ as well as neural development^26, 27^. Consistent with this, our previous discovery has shown that PLD1 negatively regulates dendritic branching in post-mitotic neurons^28^. In our previous study, we also found that PLD1 is expressed in not only early but also late developmental phase, but the function of PLD1 in the late phase remains unknown. The fact that mice lacking PLD1 exhibit deficiency in brain development and cognitive function^29^ highly suggests that PLD1 may regulate the development of dendritic spines. The growth and maturation of dendritic spines require the neurons to provide enough lipids for the rapid and significant increase in membrane and to clear the obstacles such as extracellular matrix for the dendritic spines to grow. In hippocampus, PLD1 has been reported to be expressed mainly in neurons and regulate the outgrowth of mossy fibers by stimulating the secretion of tissue plasminogen activator (tPA) which is dependent on its catalytic products PA^30, 31^. Combined with the fact that PLD1-mediated tPA signaling pathway participates hippocampal mossy fiber sprouting^31^, we believe that PLD1 may also play an important part in neuronal dendritic spine development.

To verify our hypothesis, we investigated the role of PLD1 on cultured hippocampal neurons. We discovered that overexpression of PLD1 increases the density and the area of dendritic spines, while overexpressing catalytically-inactive PLD1 (PLD1ci) functions oppositely. Consistently, knocking down PLD1 also restricts the development of dendritic spines. Further study showed that N-cadherin acts downstream of PLD1 in dendritic spine development. Finally we found that PLD1 promotes the dendritic spine development by preventing N-cadherin from being cleaved by ADAM10, suggesting a potential role of PLD1 as an important regulator and a novel therapeutic target in neurological diseases.

## Results

### PLD1 promotes the development of dendritic spines

**Figure 1:**
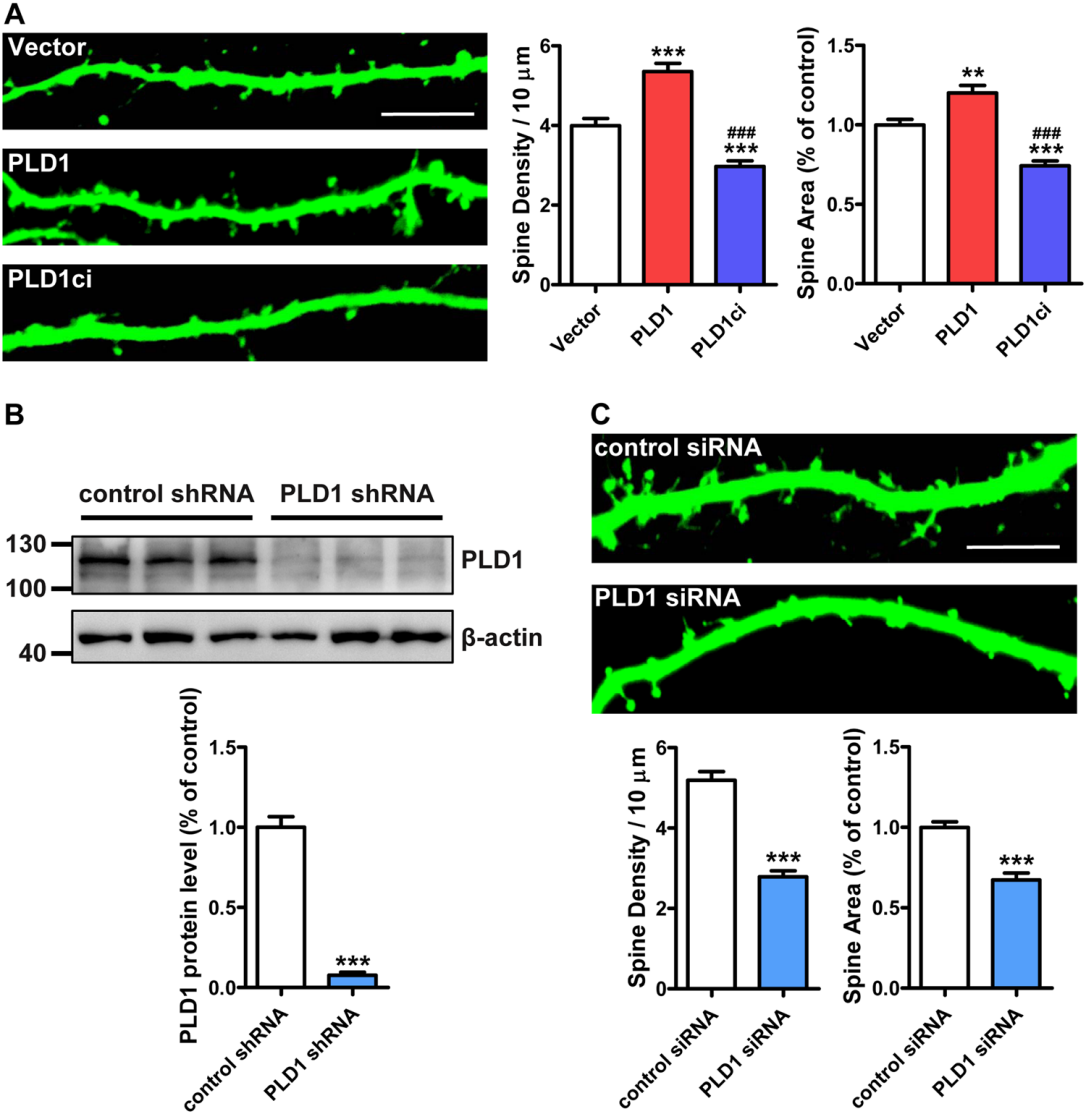
PLD1 promotes the dendritic spine development. (**A**) Representative images and quantification of the spine density and spine area in DIV15 hippocampal neurons co-transfected GFP with HA-tagged Vector, PLD1 or PLD1ci (n = 16, 12 and 16 cells, respectively in columns shown in the graphs) at DIV8. Scale bar, 10 μm. **p < 0.01, ***p < 0.001 compared with Vector, ^###^p < 0.001 compared with PLD1; one-way ANOVA with Bonferroni’s multiple-comparisons test. (**B**) Knockdown effect of endogenous PLD1 by lentivirus containing PLD1 shRNA in cortical neurons. n = 3, ***p < 0.001, unpaired *t*-test. (**C**) Representative images and quantification of the spine density and spine area in DIV15 hippocampal neurons co-transfected GFP with control siRNA or PLD1 siRNA (n = 16 and 15 cells, respectively) at DIV8. Scale bar, 10 μm. ***p < 0.001, unpaired *t*-test.

First, we used the *in vitro* cultured hippocampal neurons to explore the function of PLD1 in dendritic spine development. The neurons were transfected with HA-tagged empty vector (Vector), PLD1 and PLD1ci along with GFP to show the morphology of neurons at DIV (days *in vitro*) 8 and analyzed at DIV 15, which is the time window for dendritic spine development^32, 33^. The results showed that compared to the empty vector control, overexpressing PLD1 in hippocampal neurons induced the growth and maturation of dendritic spines as measured by their density and area (Fig. 1A). However, when overexpressed with PLD1ci, both the density and the area of dendritic spines were significantly decreased (Fig. 1A). These data imply a positive role of PLD1 in dendritic spine development. In order to testify the function of endogenous PLD1, we employed RNAi (RNA interference) approaches on cultured neurons. Lentivirus expressing short hairpin RNA and small interfering RNA targeting the same sequence of PLD1 (PLD1 shRNA and PLD1 siRNA) were constructed and applied respectively on cortical neurons and hippocampal neurons. Western blots from cultured cortical neurons infected with the lentivirus of control shRNA or PLD1 shRNA showed that the PLD1 shRNA could effectively reduce the protein level of endogenous PLD1 in neurons (Fig. 1B), which confirms the efficiency of the selected sequence. Then we transfected the cultured hippocampal neurons with GFP plus control siRNA or PLD1 siRNA. We found that knocking down PLD1 in cultured hippocampal neurons dramatically decreased the spine density and spine area (Fig. 1C). These data indicate that PLD1 promotes the development of dendritic spines.

### PLD1 regulates the membrane level of N-cadherin

**Figure 2:**
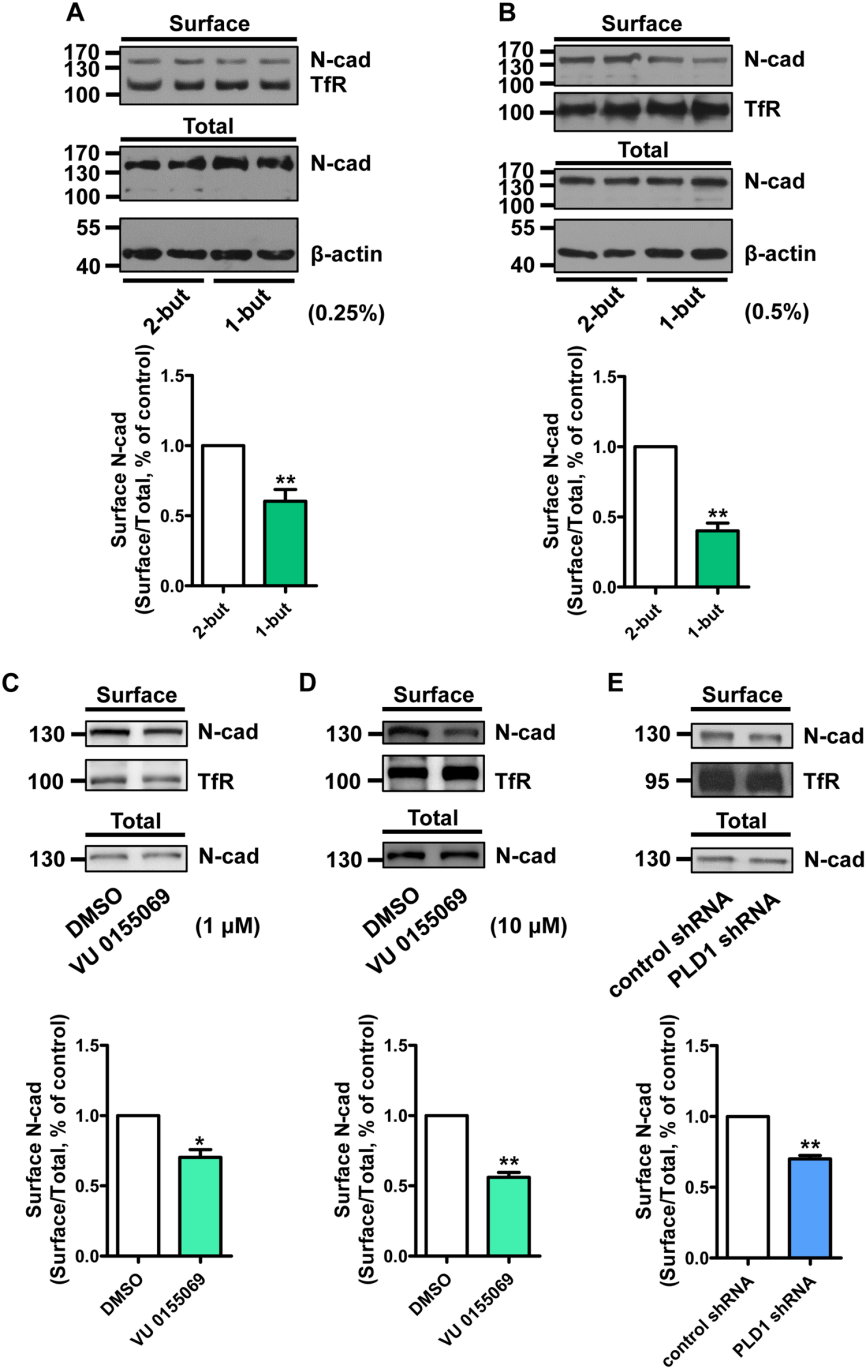
PLD1 regulates the membrane level of N-cadherin. (**A,B**) Surface biotinylation assay of membrane level of N-cadherin in cortical neurons treated with different concentration of 1/2-butanol. n = 5 (**A**) or 3 (**B**), **p < 0.01, paired *t*-test. 1/2-but, 1/2-butanol. (**C,D**) Surface biotinylation assay of membrane level of N-cadherin in cortical neurons treated with different concentration of PLD1 inhibitor VU 0155069 or vehicle control DMSO. n = 3, *p < 0.05, **p < 0.01, paired *t*-test. (**E**) Surface biotinylation assay of membrane level of N-cadherin in cortical neuron infected with lentivirus containing control shRNA or PLD1 shRNA. n = 3, **p < 0.01, paired *t*-test.

In order to form a functional synapse, the dendritic spines must match precisely with the presynaptic terminals^34^. The generation of this connectivity pattern requires the homophilic interaction of cell adhesion molecules (CAMs) located at both the axonal terminal and the dendritic spines^34^. N-cadherin, one of the important CAMs in the nervous system, has been reported to locate at the newly formed dendritic spines to mediate the pre- and post-synaptic interaction^35, 36^. Since N-cadherin executes its function in synaptic development by locating at the cell membrane, we examined the potential effect of PLD1 on the membrane level of N-cadherin. We treated cortical neurons with 1-butanol, which inhibits the production of PA^37^, or 2-butanol as a control, and then did surface biotinylation assays. We found that compared to the control groups, application of 1-butanol reduced the level of membrane N-cadherin in a dose-dependent manner (Fig. 2A,B). Because the effects of 1-butanol have been reported to be unspecific^38^, we examined the effect of another specific small-molecule PLD1 inhibitor, VU 0155069^39, 40^, on the membrane localization of N-cadherin. The results showed that treatment with VU 0155069 led to decreased membrane level of N-cadherin in a dose-dependent manner as well (Fig. 2C,D). To further specifically address the role of PLD1 on membrane N-cadherin, we infected cortical neurons with lentivirus expressing either control shRNA or PLD1 shRNA, and found that knocking down PLD1 in cortical neurons also reduced the membrane level of N-cadherin (Fig. 2E). These results demonstrate that PLD1 may promote the dendritic spine development through regulating the membrane level of N-cadherin.

### PLD1 regulates the dendritic spine development via N-cadherin

**Figure 3:**
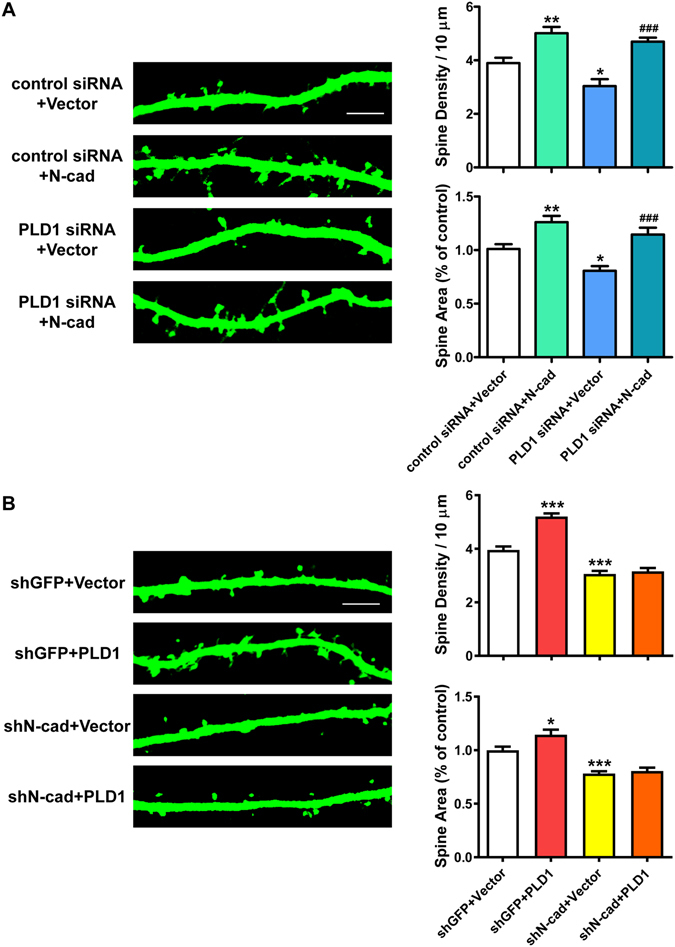
PLD1 acts upstream of N-cadherin in the dendritic spine development. (**A**) Representative images and quantification of spine density and area of DIV15 hippocampal neurons co-transfected with control siRNA + Vector (myc-Vector), control siRNA + N-cad (myc-N-cadherin), PLD1 siRNA + Vector or PLD1 siRNA + N-cad (n = 16, 14, 15 and 14 cells, respectively) plus GFP at DIV8. Scale bar, 5 μm. *p < 0.05, **p < 0.01 compared with control siRNA + Vector, ^###^p < 0.001 compared with PLD1 siRNA + Vector, one-way ANOVA with Bonferroni’s multiple-comparisons test. (**B**) Representative images and quantification of spine density and area of DIV15 hippocampal neurons transfected with shGFP + Vector (HA-Vector), shGFP + PLD1, shN-cad + Vector or shN-cad + PLD1 (n = 17, 14, 16 and 18 cells, respectively) at DIV8. Scale bar, 5 μm. *p < 0.05, ***p < 0.001 compared with shGFP + Vector, one-way ANOVA with Bonferroni’s multiple-comparisons test. shN-cad represents N-cadherin shRNA co-expressing GFP and shGFP represents control shRNA co-expressing GFP.

The results above suggest that there might be a functional link between PLD1 and N-cadherin in the dendritic spine development. So we conducted a series of knocking down and rescuing experiments on hippocampal neurons. Co-expressing N-cadherin could effectively restore the reduced spine density and spine area caused by PLD1 siRNA (Fig. 3A). Consistently, knocking down N-cadherin by shRNA (shN-cad) led to inhibition of the dendritic spine development compared to the control group (shGFP) (Fig. 3B). However, co-overexpressing PLD1 failed to rescue the effects of knocking down N-cadherin (Fig. 3B). These data show that N-cadherin acts downstream of PLD1 in promoting the development of dendritic spines.

### PLD1 prevents the cytoplasmic cleavage of N-cadherin

**Figure 4:**
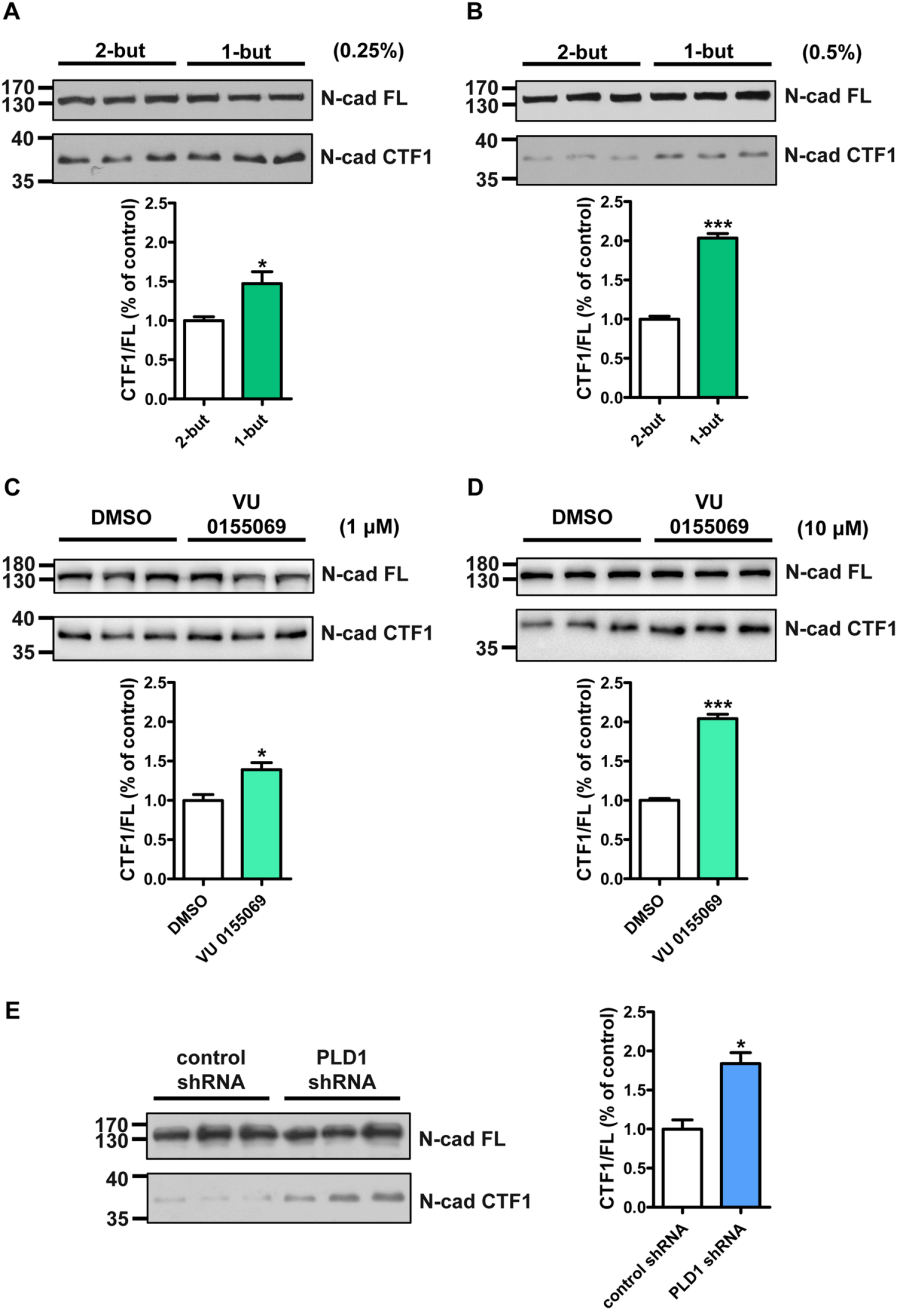
PLD1 inhibits the cytoplasmic cleavage of N-cadherin. (**A,B**) The effect of different concentration of 1-butanol treatment on the level of N-cadherin C-terminal fragment CTF1 produced by the cytoplasmic cleavage of N-cadherin in cortical neurons. n = 3, *p < 0.05, ***p < 0.001, unpaired *t*-test. N-cad FL, full-length N-cadherin. (**C,D**) The effect of different concentration of VU 0155069 treatment on the production of N-cadherin CTF1 in cortical neurons. n = 3, *p < 0.05, ***p < 0.001, unpaired *t*-test. (**E**) The effect of lentivirus containing PLD1 shRNA infection on the production of N-cadherin CTF1 in cortical neurons. n = 3, *p < 0.05, unpaired *t*-test.

**Figure 5:**
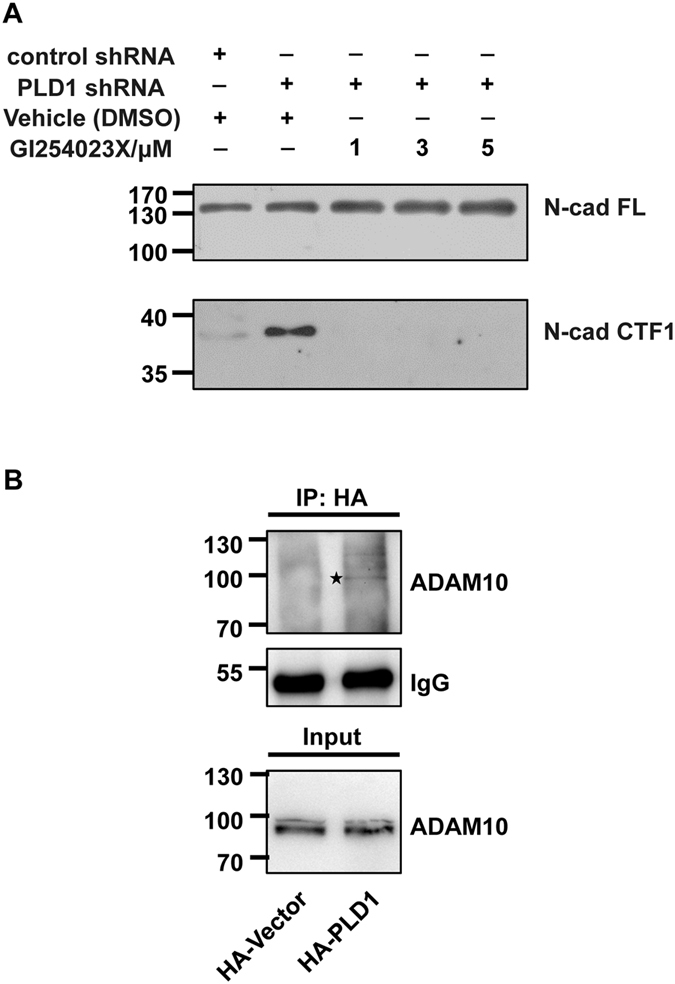
PLD1 inhibits ADAM10-mediated N-cadherin cleavage. (**A**) ADAM10 inhibitor GI254023X reversed the effect on the level of N-cadherin CTF1 caused by lentivirus containing PLD1 shRNA in cortical neurons. Three independent experiments were conducted. (**B**) Co-IP of HA-tagged PLD1 with ADAM10 in N2a cells transfected with HA-tagged PLD1. HA-Vector represents the negative control. The band of immunoprecipitated ADAM10 by PLD1 was labeled with an asterisk. IP, immunoprecipitation. Three independent experiments were conducted.

The stabilization of N-cadherin on cell membrane is dependent on its cytoplasmic interaction with β-catenin, which can be anchored to the cell cytoskeleton through α-catenin^41, 42^. It has also been reported that N-cadherin without its cytoplasmic domain acts as a dominant negative mutant to interfere the proper development of the neuronal dendrites and dendritic spines^43–45^, highlighting the necessity for the cytoplasmic domain of N-cadherin in dendritic spine development. In addition, N-cadherin can be cleaved by ADAM10, a member of the disintegrin and metalloprotease family, thus intracellularly generating a C-terminal fragment named CTF1 at dendritic spines^46–48^. Since PLD1 is a cytoplasmic protein, there is a possibility that PLD1 may regulate the membrane level of N-cadherin through affecting its cytoplasmic domain. Western blots showed that treatment of PLD inhibitor 1-butanol in the cultured cortical neurons led to an increase in the level of CTF1, and the higher concentration of 1-butanol used, the more CTF1 produced (Fig. 4A,B). The use of specific PLD1 inhibitor VU 0155069 brought about similar change in the level of CTF1 (Fig. 4C,D). Moreover, knocking down PLD1 in the cultured cortical neurons also facilitated the production of CTF1 (Fig. 4E), confirming the specific protective role of PLD1 in N-cadherin proteolysis. We further examined whether the protection of PLD1 for complete N-cadherin involves in ADAM10. The cortical neurons were infected with lentivirus expressing PLD1 shRNA or control shRNA and simultaneously treated with different concentration of GI254023X which is a specific inhibitor of ADAM10^49, 50^ or vehicle control. We found that ADAM10 inhibitor reversed the effects of knocking down PLD1 on the level of CTF1 (Fig. 5A), validating that PLD1 disrupts ADAM10-mediated cleavage of N-cadherin. Further study showed that ADAM10 forms a complex with PLD1 in N2a cells transfected with HA-tagged PLD1 compared to the HA-tagged vector control by co-immunoprecipitation (Fig. 5B). Altogether, our results demonstrate that PLD1 prevents ADAM10 from cleaving N-cadherin dependent on the catalytic activity of PLD1 and PLD1 may associate with ADAM10 to fulfill such function.

## Discussion

**Figure 6:**
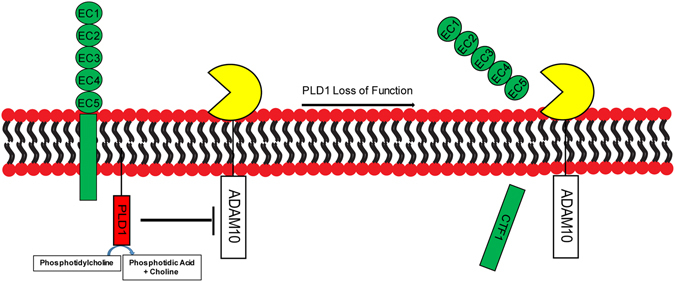
Working hypothesis. PLD1 prevents ADAM10 from cleaving membrane N-cadherin dependent on the catalytic activity of PLD1, thus maintaining the stability of membrane N-cadherin at dendritic spines. Inhibition of PLD1 activity or knocking down PLD1 loses the restriction to ADAM10 and promotes the cytoplasmic cleavage of N-cadherin.

PLD1 has been reported to function in various neuronal events, including neurite outgrowth and neurotransmitter release^31, 51, 52^. Our work first time identifies a positive role of PLD1 in the dendritic spine development, enriching the list for the roles of PLD1 play in the nervous system. We further reveal a new downstream signaling pathway for PLD1: PLD1 protects N-cadherin from being cleaved via its enzymatic activity and associating with ADAM10, thus maintaining the stability of N-cadherin on cell membrane and promoting dendritic spine development (Fig. 6).

As a member of the phospholipase D lipid-signaling enzyme superfamily, the enzymatic activity of PLD1 is important for its function. PLD1 catalyzes the hydrolysis of PC which is the most abundant membrane phospholipid, and is well-documented for its role in membrane-trafficking events^53^, implying a potential role of PLD1 in dendritic spine morphogenesis. Our previous discovery showed that PLD1 is expressed at the later stage of neural development^28^, whereas its function in later stage remains unresolved. Here we found that PLD1 promotes the dendritic spine development by regulating the membrane level of N-cadherin in neurons. Our study showed that overexpression of PLD1ci led to apparent reduction in the spine density and spine area compared to both the empty control and wild-type PLD1 (Fig. 1A). Additionally, inhibition of PLD activity by both 1-butanol and VU 0155069 dramatically depressed the membrane level of N-cadherin (Fig. 2A–D) and promoted the production of CTF1 (Fig. 4A–D). These results prove that the role of PLD1 in the dendritic spine development is also dependent on its enzymatic activity. One product of the PLD1-mediated hydrolysis is the second messenger PA, which has already been reported to promote the stability of dendritic spines^54^. Therefore, the effect of PLD1 on ADAM10 relies on the catalytic activity of PLD1.

To form a functional synapse, the axonal terminal must be connected with dendritic spines correctly, which requires the interactions between the CAMs distributed at both the pre- and the post-synaptic components^55^. As a classical CAM, N-cadherin contains five extracellular domains which mediate the homophilic interaction between cells and a cytoplasmic domain interacting with β-catenin to link with the cytoskeleton system or to mediate the signal transduction from synapse to nucleus^56, 57^. Moreover, the extracellular domain of N-cadherin has been shown to associate with GluR2 subunit^58^, which provides important insights of how the postsynaptic receptors are aligned to bind with the neurotransmitters released from presynaptic active zone. In order to achieve this, N-cadherin on the cell membrane must be anchored to the cytoskeleton system to ensure the stability of the synaptic system. N-cadherin without the cytoplasmic domain will lose its ability to stabilize the synapses. Consequently, the cleavage of N-cadherin by proteases such as ADAM10 must be precisely regulated to accommodate the growth and dynamics of the system. However, the regulation of the proteolysis of N-cadherin is still not clear.

In our study, we found that knocking down PLD1 decreased the membrane level of N-cadherin (Fig. 2C), but increased the level of N-cadherin CTF1 (Fig. 4C). We also found that PLD1 regulates the integrity of N-cadherin through affecting ADAM10-mediated cleavage of N-cadherin. Based on our co-immunoprecipitation result, we propose that PLD1 may associate with ADAM10 and increase the local concentration of PA and choline, and these products keep ADAM10 from cutting down N-cadherin through either direct inhibition or changes in the rigidity of the cell membrane around ADAM10. In this way, PLD1 may stabilize the localization of membrane N-cadherin. We also have noticed that PLD1 has been reported to inhibit the production of Aβ and promote the neurite growth^59, 60^. In our model, we do not exclude the possibility that PLD1 may regulate the membrane level of N-cadherin through affecting γ-secretase-mediated N-cadherin cleavage. Nevertheless, our data support the conclusion that ADAM10 is at least one of the targets regulated by PLD1 to modulate the membrane level of N-cadherin.

Lipid signaling has attracted much attention for their importance in diverse cell biological processes and diseases, and the lipid-modifying enzymes such as the phospholipase D superfamily also gains plenty of focus for the outlook in serving as suitable therapeutic targets^61–63^. PLD1 has been characterized for its role in cancer, pulmonary embolism and stroke^64^. Synapses have been acknowledged as the places where memories are stored and loss of synapses occurs before the onset of some neurodegenerative diseases^65^. Our work about the role of PLD1 in dendritic spine development indicates that PLD1 should also correlate with neurological disorders including neurodevelopmental diseases and neurodegenerative diseases. Combined with previous reports^59, 60^ and our results, it seems that PLD1 participates in the regulation of the function of several proteases which are responsible for membrane molecules processing. Therefore, it is promising to classify PLD1 as a pivotal molecule to modulate the activity of the proteases and neuronal development as well as a novel target for treating neurological diseases.

## Materials and Methods

### Animals

Sprague–Dawley (SD) rats were housed in a temperature- (23 ± 2 °C) and humidity- (50 ± 5%) controlled environment. The animals were maintained on a 12-h light/dark cycle with food and water *ad libitum*. All animal studies were approved by the Animal Center of the Peking University Health Science Center, and the experiments were carried out in accordance with the relevant guidelines, including any relevant details.

### DNA constructs and chemicals

HA-tagged wild-type PLD1 (PLD1) and catalytically-inactive PLD1 (PLD1ci) plasmids were generously provided by Dr. Michael A. Frohman (Stony Brook University, Stony Brook, NY). myc-tagged N-cadherin (N-cad) plasmid was kindly provided by Prof. Tanaka (Osaka University, Suita, Osaka, Japan). The pcDNA3.1 vector plasmids with corresponding tag were used as control respectively.

Lentivirus, siRNAs and shRNAs co-expressing GFP were purchased from Shanghai Genechem. The target mRNA sequence were as follows: for PLD1, 5′-CUGGAAGAUUACUUGACAA-3′, which we have verified the efficiency of >90% knockdown effect in our previous reports^28^; and for N-cadherin, 5′-GACUGGAUUUCCUGAAGAU-3′ ^66, 67^. The corresponding nonspecific sequences were used as control respectively. The vectors for shRNAs co-expressing GFP were U6-MCS-Ubi-EGFP.

1-butanol, 2-butanol, GI254023X and DMSO were purchased from Sigma Aldrich. VU 0155069 was purchased from Tocris. GI254023X and VU 0155069 were diluted in DMSO for use.

### Cell culture and transfection

Hippocampal and cortical neurons were obtained from embryonic day 18 rat embryos of either sex and plated onto 35-mm dishes coated with poly-D-lysine (Sigma Aldrich) at an appropriate density. After 4 h in plating media (10% fetal bovine serum in DMEM), the cultures were transferred to neurobasal medium supplemented with 2% B27 and 0.5 mM GlutaMAX-I (Gibco Invitrogen). Half of the medium was replaced with fresh medium every 3 days. At DIV 3, cytosine arabinoside (Sigma Aldrich) was added to the maintenance medium at a final concentration of 10 μM to inhibit glial proliferation. For morphological experiments, hippocampal neurons were transfected at DIV 8 with indicated plasmids and siRNAs along with GFP-expressing plasmid (pEGFP-N1) or shRNAs co-expressing GFP to label entire neurons using Lipofectamine-2000 (Invitrogen) following the manufacturer’s guidelines and harvested at DIV 15. In Fig. 1A, the ratio of GFP and indicated plasmids used for transfection was 1:3; in Fig. 1C, the ratio of GFP and indicated siRNAs was 1:2; in Fig. 3A, the ratio of GFP, indicated siRNAs and plasmids was 1:2:6; and in Fig. 3B, the ratio of indicated shRNAs and plasmids was 1:3. For biochemical experiments, cortical neurons were infected with lentivirus or treated with reagents at DIV 6 and collected at DIV 7 or 8.

Mouse N2a cells were maintained in DMEM supplemented with 10% fetal bovine serum. The cells were transfected with Lipofectamine-2000 (Invitrogen) according to the manufacturer’s instructions.

### Analysis of neuronal morphology

Dissociated hippocampal neurons grown at low density were used to determine the morphological characteristics of the neurons. Hippocampal neurons were fixed at DIV15 in 4% paraformaldehyde and 4% sucrose in PBS for 20 min at room temperature, mounted on slides, and imaged using an Olympus confocal laser scanning microscope (FV1000) with a 60X (NA 1.42) objective at 3X zoom. Spine stacks were acquired at 0.35-μm z-intervals to image the entire thickness of the dendrite. Measurement and analysis of the images were performed using Image Pro Plus (Media Cybernetics). Spine density was presented as average number per 10 μm of dendrites. For spine area, the experimental groups were normalized to the control groups.

### Western blots

Cell cultures were washed with ice-cold PBS, lysed in ice-cold lysis buffer (50 mM Tris-HCl, pH 7.5, 250 mM NaCl, 10 mM EDTA, 4 mM NaF, 0.5% NP-40, 1 mM PMSF) and centrifuged at12,000 × *g* at 4 °C for 5 min to extract the protein in the supernatant. The concentration of protein was measured with a BCA assay kit (Pierce). Equal amounts of samples were denatured and subjected to SDS-PAGE. After separation, proteins were transferred to nitrocellulose membranes (Pall). The membranes were blocked with 5% nonfat milk in TBST (25 mM Tris-HCl, pH 7.4, 137 mM NaCl, 2.7 mM KCl, and 0.05% Tween 20) for 1 h at room temperature and incubated with primary antibody overnight at 4 °C. After washing with TBST three times, the membranes were incubated with horseradish peroxidase (HRP)-conjugated secondary antibody (Sigma Aldrich & Origene) overnight at 4 °C, washed again and finally were developed with ECL solutions (Santa Cruz Biotechnology & Millipore). The immunoreactive bands were scanned and analyzed quantitatively by densitometry with Quantity One (Bio-Rad). The primary antibodies used in Western blots were rabbit polyclonal anti-PLD1 (Cell Signaling Technology), mouse monoclonal anti-N-cadherin 3B9 (Invitrogen), rabbit monoclonal anti-ADAM10 (Abcam), mouse monoclonal anti-human TfR (Invitrogen) and mouse monoclonal anti-β-actin (TA-09; Origene).

### Cell surface biotinylation assay

Cell cultures were washed with ice-cold PBS (pH 8.0) and then incubated for 45 min at 4 °C with 500 μg/ml EZ-Link Sulfo-NHS-SS-biotin (Pierce) to biotinylate surface proteins. After quenching with PBS containing 100 mM glycine and washing with PBS (pH 7.4), the cells were lysed with RIPA lysis buffer (TBS/1% Triton X-100/0.1% SDS). The supernatants from the cell lysates were incubated with Ultralink Plus immobilized streptavidin beads (Pierce) overnight at 4 °C to capture biotinylated surface proteins. After washing the beads six times with RIPA buffer, the bound proteins were eluted by boiling for 5 min with SDS-PAGE sample buffer and were analyzed by Western blot.

### Immunoprecipitation

Protein extracts from transfected N2a cells were prepared as for Western blots. Extracts containing 400–500 μg of protein were incubated with rabbit polyclonal anti-HA tag antibody (1:100) or normal rabbit IgG (Santa Cruz Biotechnology) at 4 °C for 3 h prior to incubation with protein A-Sepharose CL-4B resin (GE Healthcare) overnight. The immunoprecipitates were washed six times with TBS/0.1% Triton X-100. The final pellets were boiled in SDS-PAGE sample buffer and subjected to Western blot analysis.

### Statistical analysis

Statistical analysis was performed using Prism 5.0 (Graph Pad Software). Comparisons between groups were performed using Student’s *t*-test and one-way ANOVA followed by Bonferroni’s post-hoc test. All data are presented as mean ± SEM.

### Data Availability Statement

The datasets generated during and/or analysed during the current study are available from the corresponding author on reasonable request.
